# Pathogenic *Leptospira* Species in Bats: Molecular Detection in a Colombian Cave

**DOI:** 10.3390/tropicalmed7060084

**Published:** 2022-05-27

**Authors:** Carlos Ramiro Silva-Ramos, Sandra M. Chala-Quintero, Álvaro A. Faccini-Martínez, Marylin Hidalgo, Adriana del Pilar Pulido-Villamarín, Jairo Pérez-Torres, Claudia Cuervo

**Affiliations:** 1 Grupo de Enfermedades Infecciosas, Departamento de Microbiología, Facultad de Ciencias, Pontificia Universidad Javeriana, Bogotá 110231, Colombia; cramiro-silva@javeriana.edu.co (C.R.S.-R.); sandrachala@javeriana.edu.co (S.M.C.-Q.); hidalgo.m@javeriana.edu.co (M.H.); 2 Instituto de investigaciones, Fundación Universitaria de Ciencias de la Salud—FUCS, Bogotá 110231, Colombia; afaccini@fucsalud.edu.co; 3 Servicios y Asesorías en Infectología – SAI, Bogotá 110231, Colombia; 4 Committee of Tropical Medicine, Zoonoses and Travel Medicine, Asociación Colombiana de Infectología, Bogotá 110231, Colombia; 5 Departamento de Microbiología, Facultad de Ciencias, Unidad de Investigaciones Agropecuarias (UNIDIA), Pontificia Universidad Javeriana, Bogotá 110231, Colombia; adriana.pulido@javeriana.edu.co; 6 Laboratorio de Ecología Funcional, Facultad de Ciencias, Unidad de Ecología y Sistemática (UNESIS), Pontificia Universidad Javeriana, Bogotá 110231, Colombia; jaiperez@javeriana.edu.co

**Keywords:** *Leptospira*, leptospirosis, bats, Colombia

## Abstract

Leptospirosis is caused by pathogenic *Leptospira* spp., which can be found in nature among domestic and wild animals. In Colombia, the Macaregua cave is known for its bat richness; thus, because bats are reservoir hosts of human microbiological pathogens, we determined if the Macaregua cave bats harbored *Leptospira* in the wild. A total of 85 kidney samples were collected from three bat species (*Carollia perspicillata*, *Mormoops megalophylla*, and *Natalus tumidirostris)* to detect *Leptospira* spp. The *16S rRNA* gene was targeted through conventional PCR and qPCR; in addition, the *LipL32* gene was detected using conventional PCR. Obtained amplicons were purified and sequenced for phylogenetic analysis. The *Leptospira* spp. *16S rRNA* gene was detected in 51.8% bat kidneys, of which 35 sequences were obtained, all clustering within the pathogenic group. Moreover, 11 sequences presented high-identity-values with *Leptospira* *noguchii*, *Leptospira* *alexanderi*, *Leptospira* *borgpetersenii*, *Leptospira* *kirschneri*, and *Leptospira* *mayottensis*. From the *16S rRNA* *Leptospira* spp.-positive population samples, 28 amplified for the *LipL32* gene, and 23 sequences clustered in five different phylogenetic groups. In conclusion, we detected the circulation of different groups of *Leptospira* spp. sequences among cave bats in the wild; some sequences were detected in more than one bat specimen from the same species, suggesting a conspecific transmission within the cave.

## 1. Introduction

*Leptospira* (order Spirochaetales, family Leptospiraceae) is a bacterial genus which comprises a group of long, thin, and flexible Gram-negative spirochetes [1,2]. Taxonomically, *Leptospira* species are classified as pathogenic, intermediate, and nonpathogenic or saprophytic species [2]. Pathogenic *Leptospira* spp., particularly *Leptospira interrogans* and its serovars, cause leptospirosis, a neglected re-emerging infectious disease, the most widespread zoonosis in the world, and one of the etiologies of acute undifferentiated febrile illness [1,3]. The course of leptospirosis can be mild and self-limited; however, in some cases, a severe presentation with renal and liver failure (Weil’s disease) and a fatal form of the disease can be developed, including life-threatening cases [4]. Leptospirosis is considered a public health problem that is endemic in tropical regions of the world’s poorest countries. It affects mostly adult males between 20 and 49 years of age; annually, approximately 1.03 million cases occur worldwide, of which 58,900 have a fatal outcome [5].

Leptospires are maintained in nature due to persistent kidney infections among wild and domestic animals, which act as reservoir hosts and shed the bacteria in the urine for a long period [6]. Although rodents are the most important reservoirs of pathogenic *Leptospira* spp., other mammals including dogs, cats, ruminants, reptiles, birds, and bats have been proven to serve as *Leptospira* spp. carriers [6]. Hence, the study of the role of different wild animal species in the eco-epidemiology of a great number of infectious diseases has gained interest. Furthermore, the habitat or wild animals is continuously lost and fragmented due to repeated human intervention (e.g., rapid intensification of agriculture, socioeconomic change, and accelerated urbanization), which may increase the risk of contact of humans and domestic animals with wild species, due to their greater proximity [7].

Bats (order Chiroptera) represent the most diverse group of mammals and are among one of the most affected wild animals by human interventions. After humans and rodents, they are the group with the largest number of individuals, populating all continents except Antarctica. They inhabit a wide variety of natural (e.g., forests, caves) and artificial (e.g., abandoned houses, under bridges) environments [8]. Additionally, they play an important role for the natural ecosystem with functions including fertilization, pollination, seed dispersal, and arthropod population control [9]. Bats are very social, forming large colonies composed of a great number of individuals of the same or different species [10], and they have a wide variety of eating habits depending on the species (e.g., insectivores, frugivores, carnivores, hematophages) [8]. Bats are unique mammals as they have exclusive features, such as flight capacity and longevity [8]. Additionally, their immune system has reduced activity during rest and hibernation, allowing them to harbor some of the most pathogenic infectious agents without developing the disease (e.g., Rabies virus, Hendra virus, Nipah virus) [11,12,13].

Although bats are reservoir hosts of important human pathogens and may play a role as carriers of a great number of microorganisms [12,14], they also play critical ecological functions for the natural environment, which can be affected if bats are considered plagues [9]. Thus, worldwide networks have been established for bat conservation and their natural habitat [15]. In Latin America, in 2007, the Red Latinoamericana y del Caribe para la Conservación de los Murciélagos (Latin American and Caribbean Bat Conservation Network (RELCOM)) was created, which promotes regional consolidation of bat conservation, to unite efforts among partners [16]. In Colombia, of all known bat species, 217 have been described in the national territory, of which eight areas and sites are important for bat conservation according to RELCOM [17], where, to date, the Macaregua cave is considered the cave with the highest bat richness, as it harbors at least 10 different bat species (*Anoura caudifer*, *Carollia perspicillata*, *Dermanura bogotensis*, *Desmodus rotundus*, *Glossophaga soricina*, *Micronycteris megalotis*, *Micronycteris schmidtorum*, *Mormoops megalophylla*, *Myotis nigricans*, *Natalus tumidirostris*) of which three (*C. perspicillata*, *M. megalophylla*, *N. tumidirostris*) inhabit the cave permanently [18,19].

Previously, bats from diverse Colombian regions have been proven to be hosts for different *Leptospira* species, with some identified as pathogenic for humans and animals [20,21,22]. However, globally, the role of bats in *Leptospira* spp. maintenance and spread is still unknown. Considering that the Macaregua cave is recognized as the cave with the highest number of bat species, which has recently been gaining importance for research and as an ecotourism site, we aimed to determine if these bats harbor *Leptospira* spp. In addition, we wanted to establish if bats help maintain *Leptospira* spp. in the wild, and, if so, which species are circulating among them, to shed light on the eco-epidemiology of Colombian leptospirosis.

## 2. Materials and Methods

Bats from three different species (*C. perspicillata*, *M. megalophylla*, and *N. tumidirostris*), were captured inside the Macaregua cave in September 2014, June 2015, and October 2018. This cave is located in Las Vueltas village, Municipality of Curití, Santander Department, in the occidental slopes of Andes Mountains, Colombia (06 39′36″ N; 73 06′32″ W, 1565 m elevation) [19].

Permits for bat capture and sampling were obtained from the “Ministerio de Ambiente y Desarrollo Sostenible” and “Autoridad Nacional de Licencias Ambientales, (ANLA)”, Colombia, license No. 0546. Procedures involving animals were approved by the Ethics and Research Committee from the Faculty of Sciences of “Pontificia Universidad Javeriana” (ID 5696). Bats were collected using mist nets, and i.m. injection of 20 mg/kg ketamine was used to anesthetize the animal, which was then euthanized. Organs (liver, kidney, and intestine) were collected, placed in 70% ethanol, and stored at 4 °C until processed. All captured bats were sexed, weighed, and taxonomically identified using conventional morphological keys as previously reported [23]. All tissue samples used in the present study were stored at the Molecular Parasitology laboratory (Pontificia Universidad Javeriana)

DNA was extracted from 25 mg of bat kidney using the High Pure PCR Template Preparation kit according to the manufacturer’s instructions (Roche diagnostics, Mannheim, Germany). After each extraction procedure, to evaluate DNA sample integrity and rule out inhibitor presence, DNA was quantified, and its quality was evaluated with conventional PCR targeting a 940 bp fragment of the gene encoding cytochrome b (*cytB*) using CytB Uni-F and CytB Uni-R primers (Table 1) according to previously reported procedures [24]. Two negative controls were included: reaction control (sterile water added in the room where the reaction mixture was prepared) and gray control (sterile water added in the room where the DNA sample was added to the reaction mixture). In addition, a genomic bat DNA was included as a positive control. Subsequently, amplified products were visualized in a 1% agarose stained with SYBR^TM^ Safe DNA gel Stain (Invitrogen, Waltham, MA, USA).

To determine the presence of leptospiral DNA, a genus-specific real-time PCR (qPCR) targeting 331 bp of the *16S rRNA* (*rr2*) gene, using primers Lep1 and Lep2 (Table 1) and PowerUp™ SYBR™ Green Master Mix (Applied Biosystems, Austin, TX, USA), was employed [25]. To avoid unspecific amplifications, only samples with cycle threshold (Ct) values ≤ 35 were considered positive. Positive qPCR samples for leptospiral *16S rRNA* gene were amplified by a conventional PCR method using the same primers. Amplicons were evaluated in a 1% agarose gel run by electrophoresis and stained with SYBR^TM^ Safe DNA Gel Stain (Invitrogen, Waltham, MA, USA). For both procedures, two negative controls (similar to those used in the *cytB* protocol) and a positive control (*Leptospira* spp.genomic DNA) were used for all reactions. Positive *16S rRNA* gene samples were send for sequencing.

To detect DNA from pathogenic *Leptospira* spp., positive *16S rRNA* samples were evaluated with a conventional PCR protocol to amplify an amplicon of 423 bp of the major outer-membrane lipoprotein (*LipL32*) gene using the LipL32-270-F and LipL32-692-R primers (Table 1) [26], which has been described as an important virulence factor present in all pathogenic leptospires [27]. Again, positive and negative controls from the *16S rRNA* protocol were included in all PCR reactions. PCR products were evaluated using electrophoresis in a 1% agarose gel followed by staining with the SYBR^TM^ Safe DNA Gel Stain (Invitrogen, Waltham, MA, USA). Positive samples for the *LipL32* gene were sent for sequencing.

Amplicons were purified using a Wizard^®^ DNA Clean-Up System kit (Promega, Madison, WI, USA) and then bidirectionally sequenced employing a 3500 Genetic Analyzer (Applied Biosystems, Foster City, CA, USA). The forward and reverse sequences were assembled, edited, and compared with reference sequences available in GenBank after Clustal algorithm alignment. Successfully sequenced *16S rRNA-* and *LipL32*-positive samples were further analyzed by phylogenetic analysis using the neighbor-joining method [28], and the evolutionary distances were computed using the Kimura two-parameter method [29], with 1000 bootstrap replicates. All positions containing gaps and missing data were eliminated, and analyses were conducted in MEGA software, Version 7 [30].

## 3. Results

### 3.1. Detection of Leptospira spp.

A total of 85 kidney samples from three bat species were obtained, of which 35.3% (30/85) were identified as *C. perspicillata*, 35.3% (30/85) were identified as *M. megalophylla*, and 29.4% (25/85) were identified as *N. tumidirostris.* For all 85 bat kidney samples, the *cytB* gene was detected. A 51.8% (44/85) frequency of infection was established through qPCR amplifying a fragment of the *16S rRNA* gene, where bat species with insectivorous feeding habits (*M. megalophylla* and *N. tumidirostris*) presented the highest frequency of infection (Table 2).

**Figure 1:**
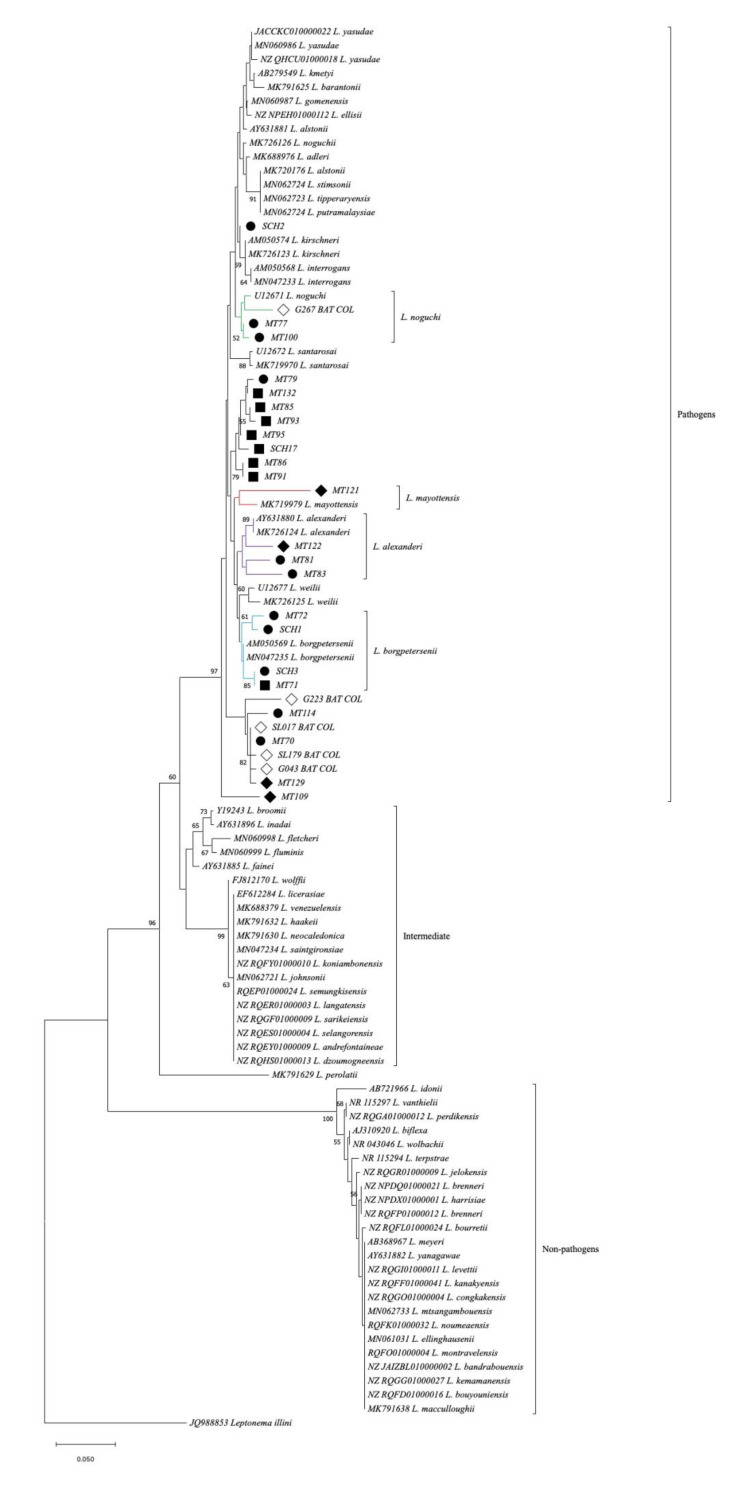
*Leptospira* spp. *16S rRNA* sequence-based phylogenetic tree detected in bats. Sequences retrieved from this study are indicated by symbols: black circles from *N. tumidirostris*, black squares from *M. megalophylla*, and black rhombuses from *C. perspicillata.* The GenBank numbers from the reference sequences are indicated in brackets, and the *Leptospira* spp. sequences obtained from previous Colombian bat species studies are indicated by white rhombuses [21]. The *Leptospira* species and their groups are listed to the right of each branch.

Out of the 44 *16S rRNA* positive samples, 35 good-quality sequences were obtained, of which 23 were different and exhibited an overall identity of 77.27% to 99.3%. Phylogenetic analysis of the partial *16S rRNA* gene reference sequences showed a proper classification into pathogenic, intermediate, and nonpathogenic subgroups. All *Leptospira* sequences obtained from the Macaregua cave clustered within the pathogenic species group (Figure 1), and 12/44 sequences displayed identity values between 85.56% and 99.3% with reference sequences of *Leptospira* currently described species, clustering with the following species: *Leptospira noguchii* (4/44), *Leptospira alexanderi* (3/44), *Leptospira borgpetersenii* (4/44), and *Leptospira mayottensis* (1/44) (Figure 1 and Table 3).

### 3.2. Detection of Pathogenic Leptospira spp.

**Figure 2:**
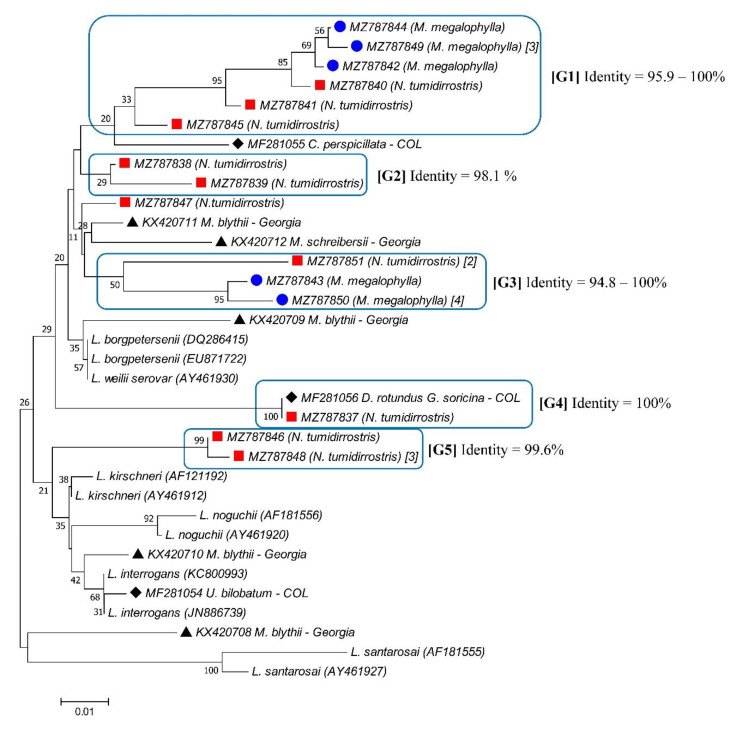
*LipL32* gene sequence-based phylogenetic tree for *Leptospira* spp. detected in bats. The sequences retrieved in this study are indicated by symbols: blue circles from *M. megalophylla* and red squares from *N. tumidirostris*; numbers in brackets in MZ787849, MZ787850, MZ787851, and MZ787848 sequences represent the number of sequences obtained from bat specimens; sequences without a particular number in brackets depict that only a sequence was obtained. GenBank numbers from reference sequences are indicated in brackets, and *Leptospira* spp. sequences obtained from bat species from previous studies are indicated by black rhombuses and black triangles [21,31]. *Leptospira* groups and identity percentages between sequences for each group are illustrated.

A total of 28 of 44 (63.6%) *16S rRNA*-positive samples amplified for the *LipL32* gene (Table 2). From these, 23 good-quality sequences were obtained, representing five groups with identity values between 94.8% and 100% (Figure 2). Groups 1 and 3 displayed the most diversity, as both groups were constituted by *Leptospira* sequences obtained from bat specimens of different species. Group 1 was made up of five sequences recovered from *M. megalophylla* and three sequences recovered from *N. tumidirostris*. Moreover, group 3 was made up of five sequences obtained from *M. megalophylla* and two sequences obtained from *N. tumidirostris*. Groups 2 and 5 included *Leptospira* sequences recovered from the same bat species (*N. tumidirostris* and *M. megalophylla*, respectively). Lastly, group 4 was constituted by only one sequence recovered from *N. tumidirostris*, which had a 100% identity with one sequence obtained from *D. rotundus* and *G. soricina* bat species captured in the department of Córdoba, Colombia [21]. Additionally, one sequence (MZ787847) obtained from *N. tumidirrostris* did not cluster with any of the other *LipL32* sequences from bats of the Macaregua cave; however, this sequence was closely related to two sequences recovered from bats captured in Georgia [31], presenting 97.4% and 96.8% identity values with KX420711 (isolated from *Myotis blythii*) and KX420712 (isolated from *Miniopterus schreibersii*), respectively.

## 4. Discussion

The present study is a contribution to shed light on the role bats play in the leptospirosis eco-epidemiology in Colombia. Three previous studies have highlighted the fact that bats are potential *Leptospira* spp. reservoir hosts in Colombia [20,21,22]. The first study was performed by Victoria et al. in 2018, who described a 26% frequency of infection determined by conventional PCR targeting the *LipL32* gene for two bat species (*Lonchophylla fornicata* and *Eumops nanus*) captured in schools from urban areas of Sincelejo municipality, department of Sucre [20]. One year later, Mateus et al. determined a 26.9% *Leptospira* positivity in stored samples of six bat species (*C. perspicillata*, *G. soricina*, *Dermanura phaeotis*, *Uroderma bilobatum*, *D. rotundus*, and *Lophostoma silvicum*) captured from tropical dry forest areas the department of Córdoba using conventional PCR targeting both genes evaluated in our study [21]. More recently, Monroy et al. evidenced a frequency of infection of 9.7% among six bat species (*C. perspicillata*, *Dermanura rava*, *G. soricina*, *Molossus molossus*, *Artibeus planirostris*, and *Uroderma convexum*) captured in the Urabá antioqueño region (Antioquia department), using conventional PCR targeting the *16S rRNA* gene [22]. Our study is the first in Colombia in which bats were collected from a cave system. We describe a 51.8% positivity for *Leptospira* infection in bats from three species captured in the Macaregua cave, department of Santander, Colombia; the highest frequency of infection was evidenced in *N. tumidirostris* bat species (64%) (Table 2). The higher frequency of infection in the present study versus previous studies may be due to the use of qPCR as a screening method, which has greater detection sensitivity when compared with conventional PCR.

It is known that at least four described *Leptospira* spp. have been detected in bats, namely, *L. interrogans*, *L. borgpetersenii*, *L. kirschneri*, and *Leptospira fainei* [32]. However, several yet-undescribed genetic clades have also been detected from a great number of bat species [32,33]. In our study, all the *Leptospira* sequences clustered among the pathogenic group. We identified *Leptospira* sequences highly similar with two aforementioned *Leptospira* spp. (*L. borgpetersenii* and *L. kirschneri*). In addition, we identified sequences highly similar to *L. noguchii* and *L. alexanderi,* which have already been detected in bats from another region in Colombia [22], as well as one sequence highly similar to *L. mayottensis*, a *Leptospira* sp. that has not been previously identified from bats worldwide. However, some sequences did not match with any of the reference sequences, which may indicate that the diversity of *Leptospira* spp. in bats is still underexplored.

We also evaluated the *LipL32* gene, whose sequence and expression are highly conserved among pathogenic *Leptospira* spp. The *LipL32* gene encodes for a protein of the same name, known as the most abundant and immunogenic lipoprotein of the outer membrane of *Leptospira*, considered an important virulence factor in the pathogenesis of leptospirosis [34]. Several *LipL32* sequences were identified in the present study. After phylogenetic analyses, different groups of *Leptospira* spp. were found to be circulating among bats from the Macaregua cave. Certain sequences were detected in more than one bat specimen of the same species (e.g., MZ787848, MZ787849, MZ787850, and MZ787851), which suggests that transmission of leptospires between bats of the same species takes place in the Macaregua cave. However, sequences were not obtained from bats of different species; thus, according to the present study *Leptospira* spp. transmission between bats of different species from the Macaregua cave cannot be confirmed. *LipL32* sequences were found only in two of the three bat species sampled, *LipL32* sequence was not detected in *C. perspicillata*. Although the phylogenetic tree constructed with *16s rRNA* gene illustrated that all sequences obtained from the three bat species grouped in the pathogenic leptospire clade, it is probable that leptospires circulating in *C. perspicillata* bats do not carry the *LipL32* gene even though they are classified in the pathogenic *Leptospira* clade.

Bats may play a role as *Leptospira* hosts, helping to maintain and perpetuate the infection in nature. It is known that bats can shed pathogenic leptospires in urine [35], raising awareness for leptospirosis among specific populations (e.g., chiropterologists, wildlife rescue workers, speleologists, etc.), due to the risk of bat contact. The leptospire excretion through animal excreta can also generate contamination of the environment [36], representing important infection sources for wild and domestic animals, and even for humans [32]. Notwithstanding, it is necessary to state that *Leptospira* excretion through bat urine is highly variable over time, which means that they can excrete different amounts of leptospires at different times, being higher at one time and minimum at another [37]. However, bat urine excretion dynamics and how these variations occur are not yet known and need further studies.

According to RELCOM, the Macaregua cave is considered the cave which harbors the greatest number of bat species (at least nine different species) in Colombia [19]. Although this cave has been unnoticed and preserved over many years, certain human activities within the cave and surrounding areas have been increasing [19]. Accelerated urbanization, growing human population, and wildlife anthropogenic interventions have made bats lose their habitat, favoring a closer contact between humans and domestic animals with bat populations that inhabit forest remnants free of human settlements [38]. Although *Leptospira* transmission may not be direct between bats and humans, an indirect infection through contact with contaminated environmental sources and infected domestic animals might represent a great risk factor for the development of leptospirosis [32,38]. However, even though some *Leptospira* spp. from the pathogenic groups have been detected in bats, it is necessary to carry out more studies to clarify the role of bats in the maintenance and spread of *Leptospira* spp. in nature to establish appropriate and timely measures focused on the prevention of future leptospirosis outbreaks, as well as on the conservation of bats’ habitat.

## 5. Conclusions

This study describes the first molecular detection and identification of pathogenic *Leptospira* spp. in bats from a cave in Colombia. We detected the circulation of different groups of *Leptospira* spp. among bats from a wildlife cave, some of them detected in more than one bat specimen from the same species, suggesting a conspecific transmission within the cave. These data reinforce the need for surveillance of zoonotic infectious agents and their circulation among wild animals, such as bats that could be playing an important role in the eco-epidemiology of the infection. Nevertheless, it is noteworthy to take into account the important ecological role that bats play.

## Figures and Tables

**Table 1:** Primers used for *Leptospira* spp. detection.

Target	Gene	Primer Name	Primer Sequence 5′–3′
Mammal	*cytB*	CytB Uni-F CytB Uni-R	TCATCMTGATGAAAYTTYGG ACTGGYTGDCCBCCRATTCA
*Leptospira* spp.	*16S rRNA*	Lep1 Lep2	GGCGGCGCGTCTTAAACATG TTCCCCCCATTGAGCAAGATT
Pathogenic *Leptospira* spp.	*LipL32*	LipL32-270-F LipL32-692-R	CGCTGAAATGGGAGTTCGTATGATT CCAACAGATGCAACGAAAGATCCTTT

**Table 2:** *Leptospira* spp. detection according to bat species.

Family	Bat Species	Feeding Habits	Samples (*n*)	*Leptospira* spp. Detection		Frequency ^2^ (%)
				*16S rRNA* ^1^	*LipL32* ^1^	
Phyllostomidae	*Carollia perspicillata*	Frugivorous	30	11	0	36.7
Mormoopidae	*Mormoops megalophylla*	Insectivorous	30	17	14	56.7
Natalidae	*Natalus tumidirostris*	Insectivorous	25	16	14	64.0
**Total**			85	44	28	51.8

^1^*16S rRNA* qPCR and *LipL32* cPCR was performed as described in Section 2. ^2^ Frequency was determined from *16S rRNA* qPCR results.

**Table 3:** *Leptospira* species identified in Macaregua Cave bats.

Bats Samples(Code)	Bats Species	*Leptospira* Species ^1^	Sequence Reference (GenBank Number)	Identity ^2^ (%)	Frequency of Infection (%)
MT100	*Natalus tumidirostris*	*Leptospira noguchii*	U12671	85.56–99.00	9.09
MT77					
MT84					
MT134					
MT122	*Carollia perspicillata*	*Leptospira alexanderi*	AY631880 MK726124	95.4–96.7	6.81
MT83	*Natalus tumidirostris*				
MT81					
SCH3	*Natalus tumidirostris*	*Leptospira borgpetersenii*	AM50569 MN047235	89.0–99.3	9.09
MT71 MT72 SCH1					
MT109	*Mormoops megalophylla*	*Leptospira mayottensis*	MK719979	95.6	2.27

^1^ Phylogenetic identification (*16S rRNA*). ^2^ Identity between bat sequences and reference sequences.

## Data Availability

The data presented in this study are available on request from the corresponding author.
